# Factors influencing the enrollment in randomized controlled trials in orthopedics

**DOI:** 10.1016/j.conctc.2017.10.005

**Published:** 2017-10-16

**Authors:** Christopher T. Lim, Heather J. Roberts, Jamie E. Collins, Elena Losina, Jeffrey N. Katz

**Affiliations:** a Harvard Medical School, 25 Shattuck Street, Boston, MA 02115, USA; b Orthopedic and Arthritis Center for Outcomes Research, Department of Orthopedic Surgery, Brigham and Women's Hospital, 75 Francis Street, BTM Suite 5016, Boston, MA 02115, USA; c Division of Rheumatology, Immunology and Allergy, Brigham and Women's Hospital, 75 Francis Street, Boston, MA 02115, USA; d Department of Epidemiology, Harvard Chan School of Public Health, 677 Huntington Ave, Boston, MA 02115, USA; e Department of Biostatistics, Boston University School of Public Health, Crosstown Building, 801 Massachusetts Avenue, 3rd Floor, Boston, MA 02118, USA

**Keywords:** Orthopedics, Randomized controlled trials, Recruitment

## Abstract

**Background:**

Low enrollment rates are a threat to the external validity of clinical trials. The purpose of this study was to identify factors associated with lower enrollment rates in randomized controlled trials (RCTs) involving orthopedic procedures.

**Methods:**

We performed a search in PubMed/MEDLINE for RCTs that involved any orthopedic surgical procedure, compared different intraoperative interventions, were published in English in a peer-reviewed journal between 2003 and 2014, and reported the numbers of both enrolled and eligible subjects. The primary outcome was the enrollment rate, defined as the number of enrolled subjects divided by the number of eligible subjects. We used a meta-regression to identify factors associated with lower enrollment rates.

**Results:**

The combined estimate of enrollment rate across all 393 studies meeting inclusion criteria was 90% (95% CI: 89–92%). Trials in North America had significantly lower enrollment rates compared to trials in the rest of the world (80% vs. 92%, p < 0.0001). Trials comparing operative and non-operative treatments had significantly lower enrollment rates than trials comparing two different operative interventions (80% vs. 91%, p < 0.0001). Among trials comparing operative and non-operative interventions, there was a marked difference in enrollment rate by region: 49% in North America and 86% elsewhere (p < 0.0001).

**Conclusions:**

RCTs investigating orthopedic procedures have variable enrollment rates depending on their location and the difference between the interventions being studied. North American trials that compare operative and non-operative interventions have the lowest enrollment rates. Investigators planning RCTs would be well advised to consider these data in planning recruitment efforts.

## 1 Introduction

Low enrollment rates can compromise clinical trials. The percentage of eligible individuals who consent to participate in a given randomized controlled trial (RCT) has been reported to be as low as 4% [1]. As a result, trials may be inconclusive or require additional time and funding to complete [2], [3]. In addition, even for adequately powered trials, low enrollment rates pose a threat to external validity [4].

As suggested by several recent trials that have helped shape treatment recommendations for common orthopedic procedures, RCTs are pivotal for investigating the efficacy of surgical procedures or their components [5], [6], [7]. It may be challenging to enroll subjects into RCTs that randomly allocate subjects to different types of procedures, since surgery is irrevocable [8], [9]. Previous studies have identified a number of reasons why an eligible individual may decline to participate in an RCT, such as a preference for one form of therapy over another, difficulty understanding the concept of an RCT, or discomfort with the idea of being randomized [1], [10], [11], [12], [13].

Investigators have examined the patient characteristics associated with whether an eligible patient enrolls or refuses to enroll in a particular trial. Factors associated with refusal to enroll have included older age, gender, marital status, language fluency, ethnicity, vocation, and socioeconomic status [10], [12], [14], [15], [16]. To our knowledge there has been no comprehensive study carried out at the trial (not subject) level that examines enrollment rates of trials and trial characteristics associated with enrollment rates.

In this study, we calculated the enrollment rates reported in publications of orthopedic RCTs over a 12-year period and assessed for associations between enrollment rate and various trial characteristics, including features of the interventions being studied, the investigators of the studies, and the publications in which the RCTs were reported. We hypothesized that trials in which the treatment arms offer similar patient experiences (e.g., two different screws for fracture fixation) would have higher enrollment, on average, than trials that randomized patients to very different experiences (e.g., surgical operation versus physical therapy). Accordingly, we hypothesized that trials comparing operative and non-operative interventions would have the lowest enrollment rates.

## 2 Materials and methods

*1. Paper selection*: Papers were included if they were written in English, were published in a peer-reviewed journal between January 2003 and December 2014, reported an RCT of living human subjects over the age of 18, and reported both the number of eligible subjects and the number of enrolled subjects. To be included, RCTs were required to have at least one arm that involved an orthopedic surgical procedure, and the arms were required to compare different intraoperative interventions. Reports of trials comparing preoperative or postoperative interventions, such as rehabilitation protocols, were not included.

Because we wished to focus on orthopedic aspects of management, we excluded studies that compared interventions involving only injections (e.g., corticosteroid vs. saline injections). Similarly, we excluded studies of different types of anesthetics. We included trials comparing intraoperative interventions such as tourniquets, drainage, and antimicrobials if they otherwise met criteria. Manuscripts that had been retracted were excluded. We selected a 12-year period in order to adequately power our findings and minimize the risk of secular trends—that is, major shifts in subject or investigator approaches to RCTs over time.

We used PubMed/MEDLINE to search for publications of orthopedic RCT results. The search was last performed on November 4, 2015. We used the following search term to identify 6727 papers for initial screening for inclusion in our study:

“(((((“2003/1”[Date - Publication]: “2014/12”[Date - Publication])) AND English[Language]) AND Randomized Controlled Trial[Publication Type]) AND (orthoped* OR orthopaed* OR arthroplast* OR arthroscop* OR meniscect* OR “cruciate ligament” OR “rotator cuff” OR laminect* OR “spinal fusion” OR “carpal tunnel release” OR “open reduction” OR “internal fixation” OR “external fixation” OR osteotom* OR “bone grafting” OR arthrodesis OR patellect* OR capsulot* OR synovect* OR syndesmot* OR “tendon repair” OR tenodesis OR “trigger finger release” OR fasciect* OR laminect* OR discect*)) AND (humans[MeSH Terms])”.

Papers were accessed through the library systems of Harvard University and two major academic hospitals (Brigham and Women's Hospital and Massachusetts General Hospital). We excluded papers if they were not available through these three library systems.

*2. Abstraction of data*: Two investigators (C.T.L. and H.J.R.) performed the screening of papers and data abstraction. They each independently screened the same initial set of 200 papers and abstracted data from papers that met inclusion criteria, and then met to resolve discrepancies and ensure a uniform approach to excluding and including papers. Thereafter, they divided all remaining papers for screening and data collection. Any papers raising uncertainty were set aside to be resolved by the team.

For each paper that met inclusion criteria, we performed a manual data extraction to obtain the following data elements. We extracted the number of subjects screened for the trial, the number of eligible subjects, and the number enrolled. We characterized the difference between study interventions; each study was categorized as either a comparison of operative and non-operative management or as one of various comparisons of operative techniques or strategies (as shown in Appendix 1). We extracted the orthopedic subspecialty area of the clinical problem addressed by the RCT, the number of months of follow-up, and whether there was inpatient follow-up only or outpatient follow-up. In terms of investigator-related data, we extracted the number of study sites for the trial, the nationality of the first author's primary institution (by region: USA/Canada, Europe, Asia/Middle East, Australia/New Zealand, Mexico/Central America/South America, other), reported external funding sources (public, foundation, and/or industry), and the number of months of recruitment. Lastly, we extracted the year of paper publication.

*3. Characterization of included and excluded papers*: We gathered data on our screening process by recording the total number of papers screened, the number of papers excluded for each inclusion/exclusion criterion, and the number of papers included in the study. Many papers were excluded based on more than one criterion, and if so they were categorized by the most salient exclusion criterion, with two exceptions. First, papers were only categorized as “not accessible” if they otherwise met criteria for inclusion. Second, among papers that met criteria for inclusion and whose full manuscripts were accessible, papers were excluded if they did not report the number of eligible subjects.

*4. Statistical analysis*: The primary outcome variable was enrollment rate, which was calculated as the number of enrolled subjects divided by the number of eligible subjects. The secondary outcome variable was screening yield, which was calculated as the number of enrolled subjects divided by the number of screened subjects. We employed a logistic random-effects model, which incorporates properties of the logistic and binomial distributions, to model the number enrolled and thus obtain an estimate for enrollment rate. The logistic random-effects model uses the exact binomial likelihood to estimate the within-study variability. Random-effects estimated by maximum likelihood were used to account for between-study variability. We used the model to calculate within-study variability, to calculate an overall combined estimate for enrollment rate and to evaluate the effect of study-level characteristics on enrollment rate [20], [21]. This allowed us to include studies with zero cells (i.e., 100% enrollment) without requiring an ad-hoc adjustment. In our model, enrollment rate and screening rate were the dependent variables, and all other variables gathered (as described in “Abstraction of data” above) were predictor variables: orthopedic subspecialty, degree of intervention difference, inpatient only vs. outpatient follow-up, duration of follow-up, single-center vs. multi-center, nationality of first author's institution, external funding source (if reported), duration of subject enrollment, and year of publication.

Study-level variables found to be significantly associated with enrollment rate differences in bivariate analysis were examined further for interactions. Given our hypothesis that studies with patient-relevant differences in intervention would have lower enrollment rates, we planned to perform interaction analyses between intervention difference and other significant predictors.

For each year of publication, we calculated the proportion of included papers (i.e., those meeting all inclusion criteria) to papers meeting all inclusion criteria except the reporting of number of eligible subjects. We termed this result the paper inclusion rate, as a proxy for the proportion of investigators reporting enrollment rates for their RCTs. We chose to examine this result rather than the proportion of included papers to all papers screened, because other RCT characteristics (e.g., the number of trials studying anesthetic use) could affect the proportion of included papers. We then assessed for any trends in paper inclusion rate by calculating a correlation coefficient between year and the paper inclusion rate.

*5. General approach:* Our study and the reporting thereof reflect PRISMA guidelines [22].

## 3 Results

**Fig. 1:**
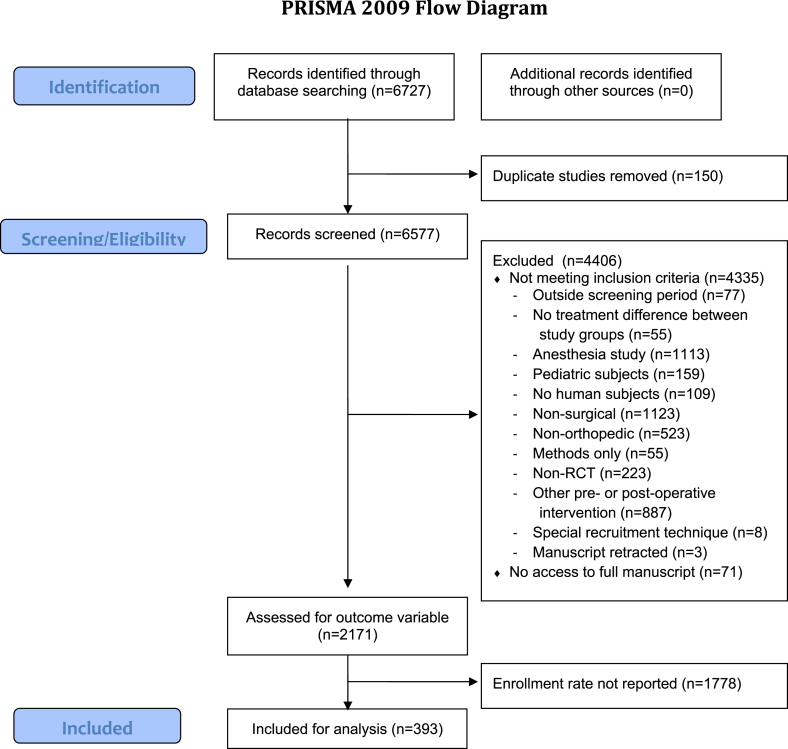
Paper inclusion flow chart.

*1. Descriptive results:* 6727 papers were screened for inclusion (See Fig. 1). 150 papers (2.2%) were duplicate citations, and were discarded. 6184 (91.9%) were excluded for not meeting the inclusion and exclusion criteria: 1778 papers (26.4%) did not report the number of eligible subjects but met all other criteria, and 4406 (65.4%) failed to meet at least one of the other criteria. 393 papers (5.8%) met all inclusion criteria, including the reporting of both the numbers of eligible and enrolled subjects, and were thus included in this study. Characteristics of the included papers are shown in Appendix 1.

The combined random-effects estimate for enrollment rate was 90.2% (95% CI: 88.9–91.5%).

**Fig. 2:**
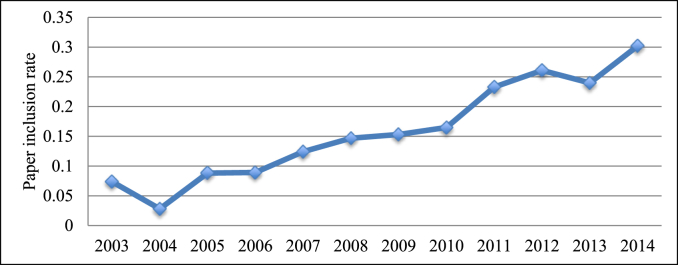
Proportion of included papers (i.e., meeting all inclusion criteria) to papers meeting all inclusion criteria except reporting of number of eligible subjects, by publication year (Pearson correlation coefficient r = 0.966, p < 0.0001).

The paper inclusion rate, a proxy for the proportion of RCT investigators reporting the enrollment rate for their trials, increased steadily over the twelve-year study period (Pearson correlation coefficient r = 0.966, Fig. 2).

*2. Intervention-related factors*: Studies in which subjects were randomized to either an operative or a non-operative intervention had significantly lower enrollment rates than studies in which subjects were randomized to one of two or more operative arms (80.0% vs. 91.3%, p < 0.0001, Table 1). We observed no significant difference in enrollment rate by follow-up type, orthopedic subspecialty, or duration of follow-up.

**Table 1:** Estimate of trial enrollment rate (with 95% confidence interval) by degree of analyzed data.

Characteristic	p-value	Enrollment rate (95% CI)
Degree of intervention difference	<0.0001	
Operative vs. non-operative		80.0% (74.0–86.0%)
Other		91.3% (90.1–92.5%)
Orthopedic subspecialty	0.2792	
Trauma		90.3% (87.3–93.3%)
Sports		88.9% (85.6–92.2%)
Reconstructive		91.4% (89.7–93.1%)
Spine		85.5% (80.0–91.0%)
Foot and ankle		89.6% (78.6–100%)
Hand		90.8% (84.6–97.0%)
Other		94.6% (85.7–100%)
Inpatient only vs. outpatient follow-up	0.0546	
Inpatient only		94.0% (90.9–97.1%)
Outpatient		89.9% (88.5–91.3%)
Duration of follow-up	0.3450	
Nationality of first author's institution	<0.0001	
USA and Canada		79.5% (74.8–84.3%)
Other		92.0% (90.9–93.2%)
Single-center vs. multi-center	0.0003	
Single-center		91.4% (90.1–92.7%)
Multi-center		84.8% (80.8–88.8%)
Public funding	0.1655	
No		90.0% (87.9–92.1%)
Yes		84.1% (79.4–88.8%)
Not reported		92.0% (90.5–93.6%)
Foundation funding	0.2959	
No		88.5% (86.1–90.8%)
Yes		88.3% (84.5–92.1%)
Not reported		92.1% (90.5–93.7%)
Industry funding	0.2868	
No		89.0% (86.7–91.3%)
Yes		88.1% (84.6–91.7%)
Not reported		91.9% (90.3–93.6%)

*Investigator-related factors*: Studies in which the first author was primarily affiliated with an institution in the United States or Canada, used as a proxy for studies performed in those countries, had significantly lower enrollment rates than studies in which the first author was primarily affiliated with an institution in another country (79.5% vs. 92.0%, p < 0.0001, Table 1). Studies that were performed at a single site had significantly higher enrollment rates than studies performed at multiple sites (91.4% vs. 84.8%, p = 0.0003, Table 1). We observed no significant difference in enrollment rate by funding source or duration of subject recruitment.

*3. Publication-related factors*: No significant difference in enrollment rate was observed by year of publication.

*4. Interactions*: After identifying nationality, intervention difference, and number of trial sites as having statistically significant effects on enrollment rate, we assessed for significant interactions between these variables. First, we evaluated the interaction between nationality and intervention difference to determine whether the effect of intervention difference on enrollment rate differed by location. We found a statistically significant interaction (p = 0.0272). For trials comparing operative and non-operative interventions, there was a marked difference in enrollment rates between studies in the US or Canada versus those in other countries (49.2% vs. 86.4%, p < 0.0001, Table 2). There was a smaller though still significant geographic difference in enrollment rates for trials comparing operative interventions, with lower rates in the US and Canada than in other countries (83.5% vs. 92.6%, p < 0.0001). In addition, the interaction between the number of sites and intervention difference was statistically significant (p = 0.0101). The difference in enrollment rates between single-site trials comparing operative and non-operative interventions and other single-site trials was not statistically significant (87.8% vs. 91.6%, p = 0.1309), whereas this difference was significant among multi-site trials (66.5% vs. 90.0%, p < 0.0001). Lastly the interaction between the number of sites and nationality was not statistically significant (p = 0.4763).

**Table 2:** Table of estimate of trial enrollment rate (with 95% confidence interval) and number of trials by degree of intervention difference and author nationality.

		Nationality	
		USA and Canada	Other
Degree of difference between interventions	Operative vs. non-operative	49.2% (32.1–66.4%)	86.4% (81.7–91.2%)
		*13 trials*	*41 trials*
	Other	83.5% (79.3–87.8%)	92.6% (91.5–93.7%)
		*70 trials*	*269 trials*

*5. Screening yield*: Of the 393 included papers, 330 (84.0%) reported the number of patients screened for eligibility. The combined random-effects estimate for screening yield was 65.9% (95% CI: 62.3%–69.5%). Significant differences in screening yield were also observed between trials comparing operative and non-operative interventions and those comparing two or more operative interventions (46.0% vs. 69.0%, p < 0.0001), between studies performed by US or Canadian investigators and those performed by investigators elsewhere (46.0% vs. 70.2%, p < 0.0001), and between studies performed at a single site and those performed at multiple sites (69.4% vs. 51.1%, p < 0.0001).

## 4 Discussion

Low rates of patient enrollment in RCTs threaten their completion and their generalizability. Previous studies have described the characteristics of and reasons stated by subjects who refuse to enroll in clinical trials, but no study has quantitatively examined the association between enrollment rate and intervention-, investigator-, and publication-related factors. In this analysis of 393 orthopedic RCTs, enrollment rate varied significantly with the degree of difference between treatment arms, region of the world in which the study was conducted, and number of sites at which the study was conducted. Of note, the difference in enrollment rates between trials comparing non-operative and operative interventions and other trials varied according to region, with a much larger enrollment rate difference in North America.

As we hypothesized, patients were less likely to enroll in studies that compared an operative to a non-operative intervention than studies that compared two or more operative interventions, regardless of the country in which the trial was performed. This may be because patients have strong preferences surrounding the irrevocable nature of the decision to undergo surgery and are reluctant to leave this decision to chance [1], [9]. Alternatively, patients may have a strong preference to undergo a surgical procedure that is the standard of care. Our finding is consistent with a prior study of 114 publicly funded RCTs in the United Kingdom, which also found lower rates of patients recruited per month per trial site among trials comparing operative and non-operative interventions [19]. However, this study examined the absolute number of patients enrolled per month, and not actual enrollment rates. Our study is the first of which we are aware to examine the proportion of patients consenting to enrollment among those eligible. Furthermore, we similarly observed a significant difference in overall screening yield between these two trial types.

Multiple factors may underlie our finding that orthopedic RCTs in the US and Canada had lower enrollment rates than those across the rest of the world. North American patients may be less willing to consent to clinical trials, perhaps because they have stronger treatment preferences or feel more authorized than their counterparts in other countries to express these preferences. It is also possible that North American investigators provide a more detailed consent process or are more willing to offer the opportunity to refuse participation, whether due to cultural differences or differences in review board requirements. Given that the most striking geographic difference in enrollment rates was in trials comparing operative and non-operative interventions, we hypothesize that patient preferences and agency to act upon these preferences played a major role. North American patients may have been willing to consent to being randomized to one of two operative treatments with seemingly minor differences, whereas they may have exercised their right to decline participation in trials that required relinquishing their choice to undergo an operation at all. Although international differences in patient recruitment have been noted in prior studies [24], further investigation of these hypotheses is warranted.

Despite wide trial-to-trial variation in approaches to recruiting a screening population, our analyses found significant differences in screening yield among factors associated with enrollment rate differences, namely author nationality, intervention difference, and site number. Investigators conducting certain types of trials may anticipate lower screening yields and thus screen larger numbers of patients to adequately power their RCTs. Given that these factors were associated with both enrollment rate and screening yield, our interpretation is that nationality and intervention difference are relevant in patients' enrollment decisions, not only in the process of confirming eligibility. The effects on the enrollment decision are strong enough to have significant effects on the overall yield of subjects from screening.

In the past two decades, an increasing emphasis on full transparency of methodology has been standardized through the Consolidated Standards of Reporting Trials (CONSORT) statements, first published in 1996 and subsequently updated in 2001 and 2010 [25], [26], [27]. Among the items recommended by the CONSORT statement is a diagram of the recruitment process that includes the numbers of screened, eligible, and enrolled subjects. In this study, we noted that the proportion of papers excluded solely on the basis of not reporting the number of eligible subjects decreased over the 12-year period, which may be in response to implementation of the CONSORT statements.

The scope of this study was limited to orthopedic RCTs; thus, we caution against generalizing to other surgical specialties. Studying the factors that are associated with enrollment rate in RCTs in other specialties may help to generalize our findings and identify other key factors that influence enrollment rates. Another limitation of this study was that we did not report on subject-related characteristics, as demographic statistics about the population of eligible individuals who declined participation in a study are seldom included in papers. In addition, we did not adjust for multiple comparisons in our analysis of factors associated with enrollment rate. Lastly, we noted that multi-center trials that compared operative and non-operative interventions had lower enrollment rates than multi-center trials comparing operative interventions, whereas this difference was much less striking for single-center trials. We are not certain about the reason for this interaction. It is possible that multi-center trials involve more complex decisions for subjects (e.g., more invasive procedures), resulting in lower enrollment.

Our results show that the country in which a trial is performed may influence enrollment rate, especially for trials comparing operative and non-operative interventions. This finding has implications beyond orthopedics that warrant further investigation. Underlying the geographic differences in enrollment rates may be cultural and economic differences pertaining to the relationship between patients and medical professionals as well as incentives to conduct or enroll in trials. As lower enrollment rates may compromise external validity, we urge more research in this area to validate these findings and devise strategies to remedy them.

## Source of funding

There was no external funding source for this study.

## Figures and Tables

**Table:** 

Paper characteristics	Number of papers (%)
Orthopedic subspecialty	
Trauma	68 (17.3)
Sports	75 (19.1)
Reconstructive	184 (46.8)
Spine	42 (10.7)
Oncology	0 (0.0)
Foot and ankle	6 (1.5)
Hand	15 (3.8)
Other	3 (0.8)
Degree of intervention difference	
Operative vs. non-operative	54 (13.7)
Other	
Different procedures	50 (12.7)
Different approaches or techniques, same procedure	80 (20.4)
Same procedure with or without additional procedure	32 (8.1)
Different materials, same procedure	79 (20.1)
Different use of intraoperative technology, same procedure	32 (8.1)
Other	66 (16.8)
Inpatient only vs. outpatient follow-up	
Inpatient only	30 (7.6)
Outpatient follow-up	361 (91.9)
Not reported	2 (0.5)
Duration of follow-up	
≤6 months	110 (28.0)
>6 months, up to 12 months	115 (29.3)
>12 months, up to 24 months	103 (26.2)
>24 months	63 (16.0)
Not reported	2 (0.5)
Single-center vs. multi-center	
Single-center	309 (78.6)
Multi-center	80 (20.4)
Not reported	4 (1.0)
Nationality of first author's institution	
USA and Canada	83 (21.1)
Europe	204 (51.9)
Asia and Middle East	75 (19.1)
Australia and New Zealand	24 (6.1)
Mexico, Central America, and South America	4 (1.0)
Other	3 (0.8)
External funding source, if reported (Not mutually exclusive categories)	
Public funding	64 (16.3)
Foundation funding	61 (15.5)
Industry funding	71 (18.1)
Duration of subject enrollment	
0–12 months	54 (13.7)
13–24 months	116 (29.5)
25–48 months	115 (29.3)
>48 months	49 (12.5)
Not reported	59 (15.0)
Year of publication	
2003	7 (1.8)
2004	3 (0.8)
2005	12 (3.1)
2006	14 (3.6)
2007	18 (4.6)
2008	27 (6.9)
2009	30 (7.6)
2010	31 (7.9)
2011	50 (12.7)
2012	67 (17.0)
2013	57 (14.5)
2014	77 (19.6)
